# Hierarchical multi-label classification based on LSTM network and Bayesian decision theory for LncRNA function prediction

**DOI:** 10.1038/s41598-022-09672-1

**Published:** 2022-04-06

**Authors:** Shou Feng, Huiying Li, Jiaqing Qiao

**Affiliations:** 1 College of Information and Communication Engineering, Harbin Engineering University, Harbin, 150001 China; 2 Ministry of Industry and Information Technology, Key Laboratory of Advanced Marine Communication and Information Technology, Harbin, 150001 China; 3 Harbin Institute of Technology, School of Electronic and Information Engineering, Harbin, 150001 China

**Keywords:** Data mining, Data processing, Gene ontology, Machine learning

## Abstract

Growing evidence shows that long noncoding RNAs (lncRNAs) play an important role in cellular biological processes at multiple levels, such as gene imprinting, immune response, and genetic regulation, and are closely related to diseases because of their complex and precise control. However, most functions of lncRNAs remain undiscovered. Current computational methods for exploring lncRNA functions can avoid high-throughput experiments, but they usually focus on the construction of similarity networks and ignore the certain directed acyclic graph (DAG) formed by gene ontology annotations. In this paper, we view the function annotation work as a hierarchical multilabel classification problem and design a method HLSTMBD for classification with DAG-structured labels. With the help of a mathematical model based on Bayesian decision theory, the HLSTMBD algorithm is implemented with the long-short term memory network and a hierarchical constraint method DAGLabel. Compared with other state-of-the-art algorithms, the results on GOA-lncRNA datasets show that the proposed method can efficiently and accurately complete the label prediction work.

## Introduction

In recent years, noncoding RNA (ncRNA) has become a hot spot. With the continuous progress of genome annotation, the results show that only approximately 1–2% of the genes in the mammalian genome are involved in the work of coding proteins, while the previously neglected non coding sequences also play vital roles in all life activities^1^. Among these noncoding sequences, a transcript type with a length of more than 200 nucleotides and an inability to encode proteins, called long noncoding RNA (lncRNA), has attracted special attention. It has been found that lncRNAs not only have rich biological functions, but also widely participate in various important physiological processes^2^.

As lncRNAs play an important role in regulating biological activities, determining the biological function of lncRNAs has also become particularly important. Although traditional experimental methods can accurately determine the functions of RNAs, these experimental methods often require high-throughput biological experiments, including cumbersome and complex sequencing and comparison^3–6^. Researchers often spend considerable time and cost labelling few functions, and the experimental results are often not processed in a timely manner or effectively.

With the rapid increase in massive bioinformatics data, an increasing number of lncRNAs have been found and labeled. However, using traditional experimental methods to determine the function of these lncRNAs obviously cannot meet the actual biological needs. To solve these problems, many researchers use computational methods to establish prediction models to predict the functions of lncRNAs^7^. Predicting lncRNA functions by computational methods can greatly reduce the time required for the annotation of lncRNA functions. As long as the predicted function is given and the scope is reduced, researchers can carry out verification experiments with direction and basis, which can not only reduce the experimental cost, but also promote the rapid development of functional genomics^8^.

Since there are few known lncRNA functions, existing studies on computational methods for predicting lncRNA functions usually use graph theory and statistics-related knowledge to establish a correlation network between lncRNAs and proteins, DNA, and other RNAs, and then based on the principle of ’guilty by association ’, compute the relevance obtained from the network to annotate functions for lncRNAs. Guo constructed a bi-coloured network whose vertices represented protein-coding and non-coding genes, and whose edges represented co-expression and protein interactions. Then, they designed a global propagation algorithm on the bi-coloured network, and computed an association score for each unannotated lncRNA, measuring how much it could be annotated with a function^9^. Zhao constructed two bi-coloured networks, each of which corresponded to a view of lncRNA gene association, and each type of genomic data was used to construct the lncRNA-gene associations^10^. Jiang used the hypergeometric test to functionally annotate a single lncRNA or a set of lncRNAs with significantly enriched functional terms among the protein-coding genes that were significantly coexpressed with the lncRNAs.He also provided a comprehensive resource for the functional investigation of human lncRNAs, LncRNA2Function^11^. The lncRNA similarity network, protein interaction network, and lncRNA-protein coexpression network were used to construct a global heterogeneous network in Zhang’s work^1^, and then the correlation coefficients between lncRNAs and proteins were determined by the bi-random walk method. Finally, the possible functions of lncRNAs were annotated in Gene Ontology(GO) terms based on the highly-ranked adjacent protein-coding genes.

**Figure 1:**
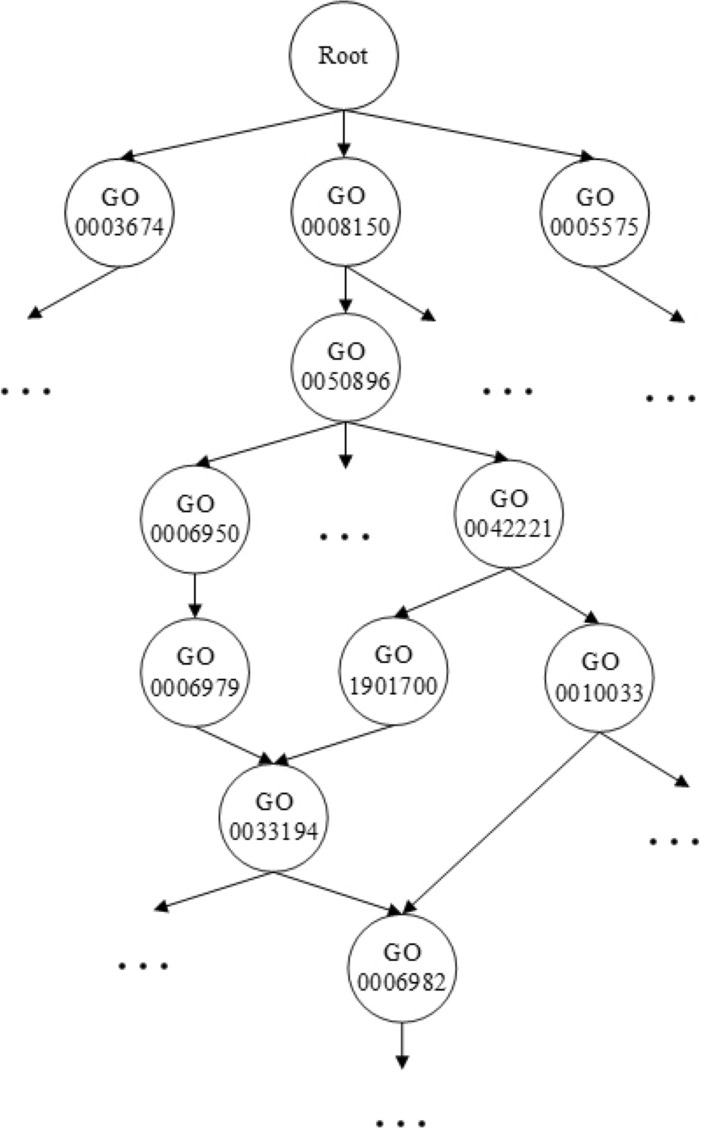
The schematic diagram of GO.

Studies show that most previous work has focused on the construction of a correlation network and the computation of the score of similarities^7^. Machine learning methods are hardly involved in the prediction stage, although Zhao^10^ pointed out that the neural network NeuraNetL2GO used in Zhang’s work^12^ could make more use of graph embedding features from networks. The deep learning architectures DBN and DNN for lncRNA identification and lncRNA-protein interaction prediction used in Yang’s method LncADeep also perform well and have demonstrated the effectiveness of deep learning methods, which are capable of learning sophisticated hidden structures in data^13^. In fact, because lncRNA function annotation generally adopts a GO scheme, its unique directed acyclic graph (DAG) structure determines that each function is not independent, but has a certain hierarchical constraint relationship. The GO resource is the world’s most comprehensive and widely used knowledge base concerning the functions of genes and gene products (proteins and noncoding RNAs). This information plays a critical role in the computational analysis of genomic and biomedical data. Statistics show that GO has been cited by over 100 000 scientific publications to date^14,15^. The GO annotation scheme organizes functional terms by the DAG structure, and each node in the DAG represents a particular function. It is believed that utilizing the relationships among GO terms would improve the prediction ability^1^. A portion of the GO resource is shown in Fig. 1^16^. All terms in the domain can be traced back to the root term, and each term may have multiple child terms or multiple parent terms. There may be many different paths to the root ontology through a different number of intermediate terms^17^. Therefore, when lncRNAs adopt a GO annotation scheme for functional annotation, lncRNA function prediction should be regarded as a hierarchical multilabel classification problem for DAG structures rather than a binary classification problem or a flat multilabel classification problem^18,19^.

To address the above issues, a hierarchical multilabel classification method based on a long short-term memory (LSTM) network and Bayesian decision theory (HLSTMBD) is proposed for lncRNA function prediction in this paper. The proposed method designs an LSTM network for all function nodes of the GO, and the information of different nodes’ relationships in the DAG is considered by Bayesian decision theory. Furthermore, the DAGLabel algorithm is utilized to ensure the hierarchical constraint of the final results. Our contributions can be summarized as follows:

First, to improve the prediction accuracy of all function nodes, the LSTM network is designed in the proposed HLSTMBD method. With the help of the depth feature extraction and mining ability of the LSTM network, the classification accuracy of each node can be improved.

Second, to fully consider and make use of the hierarchical information between parent and child nodes in the DAG structure, a mathematical classification model based on Bayesian decision theory (BD model) is constructed to change the hierarchical multilabel classification problem into a conditional risk minimization problem, which can integrate the hierarchy information and treat different prediction errors that may occur at parent and child nodes with different costs.

Third, to address the problem that prediction results become meaningless because they do not meet the hierarchical constraints, the DAGLabel algorithm is adopted to ensure the hierarchical constraint of the final results of the proposed HLSTMBD method, which can also ensure the accuracy of the final results.

The remainder of this paper is structured as follows. “Notations and definitions” section presents preliminaries on hierarchical multilabel classification. Then, the proposed method is elaborated in detail in “The proposed method” section. The experimental results are provided in “Experiment” section. Finally, the paper is concluded in “Conclusion” section.

## Notations and definitions

In this section, the notations and definitions in hierarchical multilabel classification are described first, and then Bayesian decision theory is presented.

### Hierarchical multi-label classification

Let ${\mathscr {X}}=\ {\mathbb {R}}_{p}$ be a $\ p$-dimensional input feature space, each instance can be written as a vector with $\ p$ features. ${\mathscr {L}}=\{l_1,l_2,\ldots ,l_c\}$ is a predefined class label finite set. There are *c* possible labels, where $c\ge 2$, and these labels conform to a hierarchy. $\ H$ is a predefined hierarchy, which can be a tree or a DAG. Each label in $\ {\mathscr {L}}$ corresponds to a node in $\ H$, and the label set $\ {\mathscr {L}}$ can be represented by *H*. $D=\{({\mathbf {x}}_i, {\mathbf {y}}_i) \vert i=1,2\ldots m\}$ is a training dataset, and ${\mathbf {x}}_i=(x_{i1},x_{i2},\ldots , x_{ip})\in {\mathscr {X}}$ is an instance. ${\mathbf {y}}_i=(y_{i1},y_{i2},\ldots , y_{ic})$ is the label vector of ${\mathbf {x}}_i$, and $y_{ij}=1$ means ${\mathbf {x}}_i$ has label $l_j$, and $y_{ij}=0$ means ${\mathbf {x}}_i$ does not have label $l_j$, and these labels can have multiple paths in *H*.

The DAG hierarchy can be denoted by $G=<V,E>$ with vertices $V=\{1,2,\ldots ,\vert V \vert \}$ and edges $e=(k,l)\in E,k,l\in V$. The nodes *V* represent the classes, so $V={\mathscr {L}}$ and the class $l_i$ is represented simply by the node *j* if there is no ambiguity.A direct edge $e=(i,j)\in E$ describes the hierarchical relationship that *i* is the parent node of *j*. We further denote the set of children of a node *i* as *child*(*i*), the set of its parents as *par*(*i*), the set of its ancestors as *anc*(*i*), the set of its descendants as *desc*(*i*) and the set of its siblings as *sib*(*i*).

The task of hierarchical multilabel classification is to learn a mapping function *f* from training dataset *D*, where $f: {\mathscr {X}}\rightarrow 2^{{\mathscr {L}}}$. For an unknown instance ${\mathbf {x}}_t\in {\mathscr {X}}$, the function $f(\cdot )$ can predict the label vector $f({\mathbf {x}}_t)$ of the instance ${\mathbf {x}}_t$. The mapping function $f(\cdot )$ is also called the hierarchical multilabel classifier^20^.

### The hierarchy constraint

For hierarchical multilabel classification, because of the hierarchical structure between labels, the classification result of an instance must satisfy hierarchical constraint^21^. Let ${\mathbf {y}}$ be the true label of an instance ${\mathbf {x}}$, and $y_i$ be the i-th component of ${\mathbf {y}}$. $y_i=1$ means that the instance belongs to node *i*, and ${\mathbf {y}}_{par(i)}$ means the component set of *par*(*i*) in ${\mathbf {y}}$, then the mathematical form of the hierarchical constraint for a directed acyclic graph can be expressed as:

1 $$\begin{aligned} \left\{ \begin{array}{l} y_i =1\Rightarrow \{i=0 \cup {\mathbf {y}}_{par(i)}=1\}\\ y_i =0\Rightarrow { {\mathbf {y}}_{desc(i)}=0} \end{array} \right. \end{aligned}$$

### Bayesian decision theory for HMC

Loss function^22^, also known as cost function, is used to measure the degree of inconsistency between the predicted value of the model and its real label, that is, the degree of prediction error of the model. The loss function is a non-negative real value function between the predicted value and the real value of an instance. The smaller the difference between the real value and the predicted value, the smaller the value of the loss function, and the better the model. Let $f(\cdot )$ be a classifier; the loss function of an instance ${\mathbf {x}}$ can be written as $L({\mathbf {y}},f({\mathbf {x}}))$. For a classification problem, the conditional risk $R(\hat{{\mathbf {y}}}\vert {\mathbf {x}})$ of classifying its label as $\hat{{\mathbf {y}}}$ is

2 $$\begin{aligned} \begin{aligned} R(\hat{{\mathbf {y}}}\vert {\mathbf {x}})&=\sum _{{\mathbf {y}}\in \{0,1\}^c}L({\mathbf {y}},\hat{{\mathbf {y}}})P({\mathbf {y}}\vert {\mathbf {x}}) \end{aligned} \end{aligned}$$

According to Bayesian decision theory based on the minimum risk principle^23^, for an instance ${\mathbf {x}}$, the label that minimizes conditional risk is the predictive label of the instance, therefore, the prediction of an instance’s labels becomes the following optimization problem:

3 $$\begin{aligned} \begin{aligned} \hat{{\mathbf {y}}}^{*}&=\arg \min _{\hat{{\mathbf {y}}}\in \{0,1\}^c}R(\hat{{\mathbf {y}}}\vert {\mathbf {x}}) \\&=\arg \min _{\hat{{\mathbf {y}}}\in \{0,1\}^c}\sum _{{\mathbf {y}}\in \{0,1\}^c}L({\mathbf {y}},\hat{{\mathbf {y}}})P({\mathbf {y}}\vert {\mathbf {x}}) \end{aligned} \end{aligned}$$

## The proposed method

**Figure 2:**
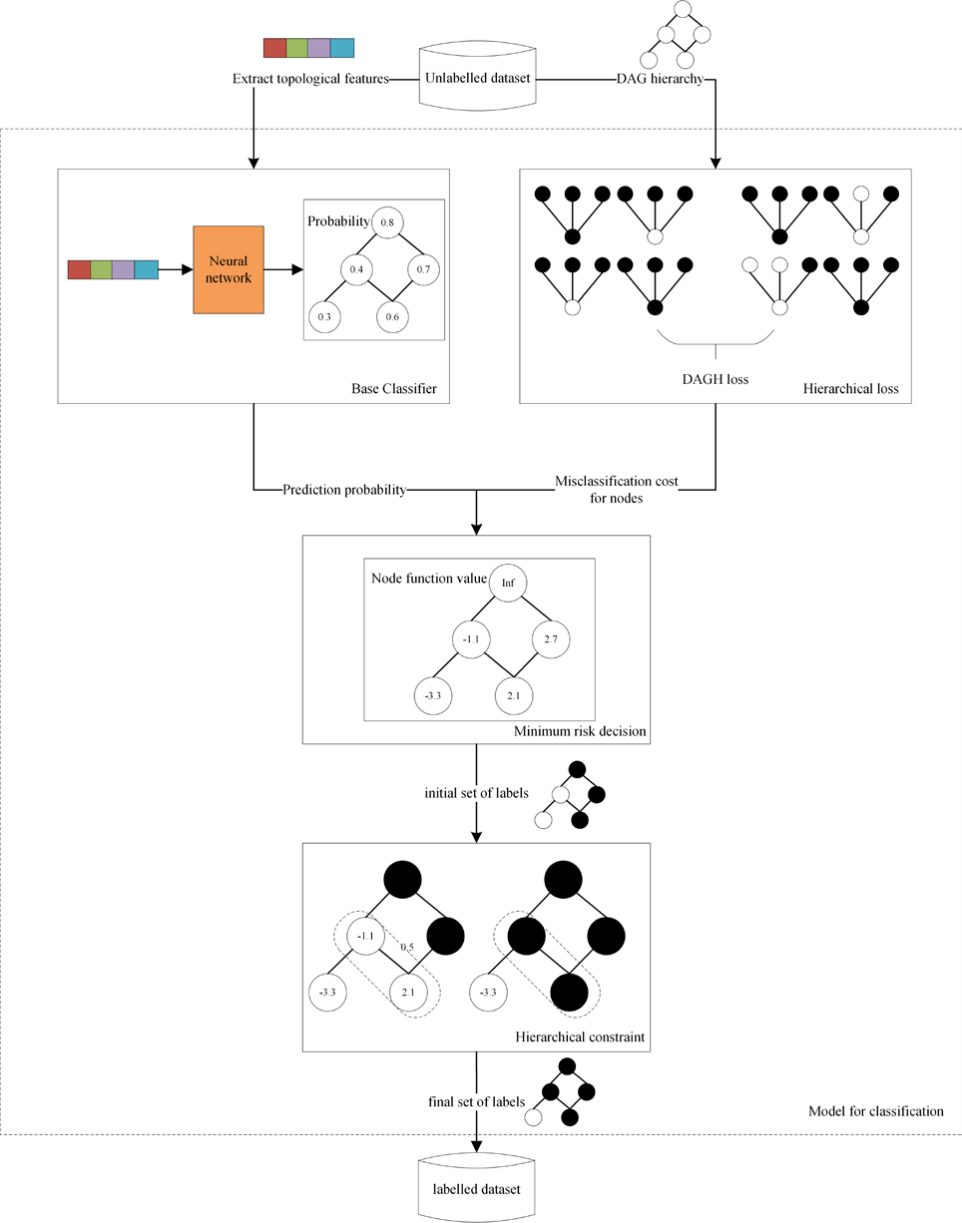
The classification process.

The framework of the proposed method can be described by Fig. 2. Considering that nodes with similar topological features tend to have similar functions, we used low-dimensional topological features as the representative vector of each node, which were extracted from the lncRNA coexpression network with the diffusion component analysis (DCA) approach as described in Zhang’s work^12^. We first use a random walk with restart algorithm (RWR) on each node to obtain a combination of the local and global topological information. The RWR algorithm is an improvement on the basis of the random walk algorithm. Starting from a certain node in the graph, each step faces two choices: randomly select the adjacent node or return to the starting node. The algorithm contains a parameter ${\mathbf {r}}$, which is the restart probability, and $\mathbf {1-r}$ represents the probability of moving to an adjacent node. After iterations, it tends to be stable, and the probability distribution obtained after the plateau can be regarded as a distribution affected by the starting node. RWR can capture both the multifaceted relationship between two nodes and the overall structural information of the graph.Then the high dimension obtained by RWR is reduced by singular value decomposition. After that, we obtain the features of each node and our classification process starts.

First, an LSTM network is designed and trained for all nodes of the DAG structure, and according to the characteristics of each instance, the predicted probability for each label is obtained. Next, the mathematical model based on Bayesian decision theory is designed to integrate the primary classification results obtained by each LSTM network. Finally, since there may be labels in the label set that violate the hierarchy constraint at this time, the hierarchical constraint algorithm DAGLabel is used to modify them to obtain the predicted label set that conforms to the hierarchical structure.

**Figure 3:**
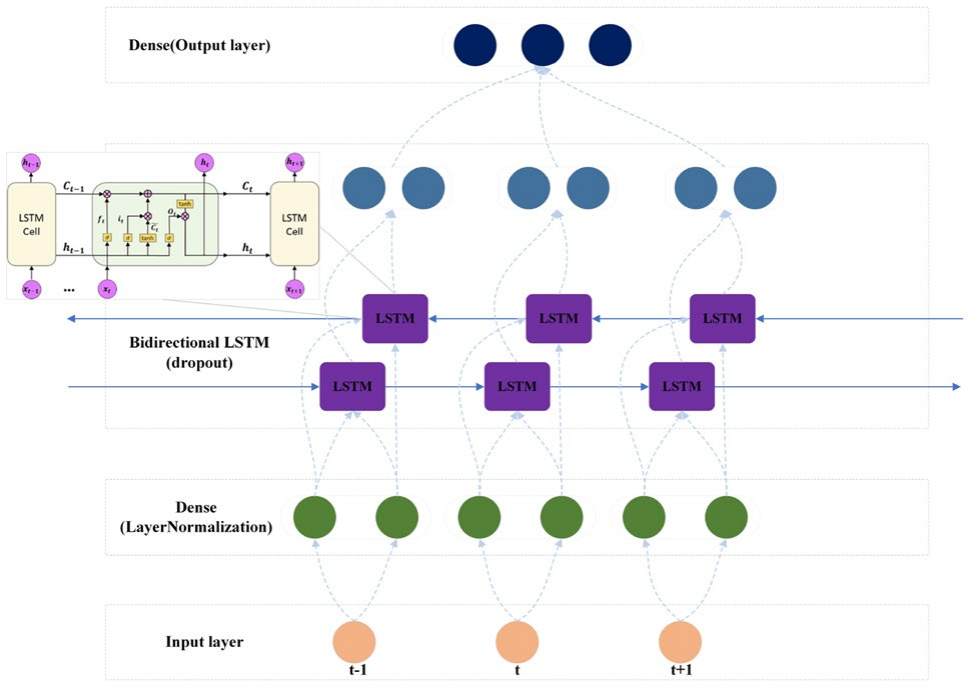
The description of the model.

The main content of the proposed HLSTMBD method includes the following three parts: the design of the LSTM network, the construction of the BD mathematical model and the design of the hierarchical constraint algorithm .

### The design of the LSTM network

In this paper, the bidirectional LSTM is chosen as the basic classifier. LSTM is a sequence-to-sequence model and is widely used in natural languange processing(NLP) fields. It is well-known for its good performance on memory function^24–26^. For our hierarchical multilabel problem, the input features can be seen as a sequence, and the output labels can also be seen as a sequence. In this case, the LSTM network can classify all labels at one time and is expected to capture implicit hierarchy information.

The model framework is described in Fig. 3. First, the dense layer is applied to extend the input dimension, and then layer normalization is used to normalize the extended features. Then the BiLSTM network is applied to capture latent relationships between instances. The BiLSTM network has two independent LSTMs. The input sequence is sent to the two LSTM neural networks in positive and reverse order for feature extraction, and the feature vector formed by splicing the two output vectors is used as the final feature expression of the instance^27^. Thus, the BiLSTM network can integrate both past and future information, which is necessary because the information in the future period of time is equally important for the prediction of current instances. To avoid overfitting, a dropout layer is added before the final classification.

After this network, we can obtain the posterior probability $p_{j}$ of each instance at node*j*, which is related to the construction of the Bayesian decision (BD) mathematical model.

### The mathematical model based on Bayesian decision theory

According to Bayesian decision theory, for DAG hierarchical multilabel classification, the optimization function can be written as:

4 $$\begin{aligned} \begin{aligned} \hat{{\mathbf {y}}}^{*}&=\arg \min _{\hat{{\mathbf {y}}}\in \Psi }R(\hat{{\mathbf {y}}}\vert {\mathbf {x}}) \\&=\arg \min _{\hat{{\mathbf {y}}}\in \Psi }\sum _{{\mathbf {y}}\in \{0,1\}^c}L({\mathbf {y}},\hat{{\mathbf {y}}})P({\mathbf {y}}\vert {\mathbf {x}}) \end{aligned} \end{aligned}$$

where $\Psi$ denotes a multilabel set that satisfies the hierarchical constraint expressed in the formula (1). It is easy to find that the set $\Psi$ is a subset of the label space, so $\Psi \subseteq \{0,1\}^c$.

Formula (4) can be seen as a mathematical model for solving DAG hierarchical multilabel classification. To complete this mathematical model, a suitable loss function should be designed first. Then, the solution to the function 4 should be derived.

#### The hierarchical loss function

To design a good loss function for the DAG hierarchical structure, the possible prediction errors in DAG hierarchical multilabel classification should first be analysed in detail.

For an instance ${\mathbf {x}}$ and a hierarchy *H*, when an instance has a prediction error at a node *i*, there are two possible cases. The first case is that the true label at node *i* is 1, but the prediction label is 0, which can be written as $y_i=1$ and ${\hat{y}}_i=0$. The other case is that the true label at node *i* is 0, but the prediction label is 1, which can be written as $y_i=0$ and ${\hat{y}}_i=1$. The logical compliment represented by $\tilde{y_i}$ is $\tilde{y_i}= 1-y_i$. Therefore, when $y_i = 1$, $\tilde{y_i} = 0$ and when $y_i = 0$, $\tilde{y_i} = 1$.

For the first case, the prediction results at its parent nodes have two cases.

*Case (a)* its parent nodes are all correctly predicted, which can be written as ${\mathbf {y}}_{par(i)}=1$ and $\hat{{\mathbf {y}}}_{par(i)}=1$.

*Case (b)* the true labels of all its parent nodes are 1, but the prediction label of at least one parent node is 0; that is, the prediction results of its parent nodes are not completely correct. This case can be written as ${\mathbf {y}} _{par(i)}=1$, and $\hat{{\mathbf {y}}}_{{par}_{p0}(i)}=0$, $\hat{{\mathbf {y}}}_{{par}_{p1}(i)}=1$, where the set of its parent nodes whose predicted value is 0 is denoted as ${par}_{p0}(i)$, and the set of remaining nodes is denoted as ${par}_{p1}(i)$.

Similar to the first case, for the second case, the prediction results at its parent nodes also have two cases.

*Case (c)* its parent nodes are all correctly predicted, which can be written as ${\mathbf {y}}_{par(i)}=1$ and $\hat{{\mathbf {y}}}_{par(i)}=1$.

*Case (d)* the prediction results of each parent node are not all correct. Define the set of parent nodes whose true values are 0 as ${par}_{t0} (i)$, and the set of remaining nodes is denoted as ${par}_{t1} (i)$, this case can be written as ${\mathbf {y}} _{{par}_{t0}(i)}=0$, and ${\mathbf {y}}_{{par}_{t1}(i)}=1$, $\hat{{\mathbf {y}}}_{par(i)}=1$.

From the above analysis, when a sample has a prediction error at a node, the possibility of the parent node’s error has four cases. Referring to the form of the loss function designed for the tree structure and integrating the information related to the possible errors into the loss function, in this paper, a loss function for a directed acyclic graph structure is proposed, namely, the DAG hierarchical loss function, which is abbreviated as the DAGH loss function.

In the DAGH loss function, each item corresponds to the parent-child node error type mentioned above. Let there be *N* nodes in a directed acyclic graph *H*, denoted as $\{0,1,2,\ldots ,N-1\}$, where the 0 node is the root node. Then, the formula of the DAGH loss function is as follows:

5 $$\begin{aligned} L_{DAGH}(\hat{{\mathbf {y}}},{\mathbf {y}})=\ell _{1}+\ell _{2}+\ell _{3}+\ell _{4} \end{aligned}$$

The specific forms of these items are as follows:

6 $$\begin{aligned} \ell _{1}&= w_{1}\sum ^{N-1}_{i=1} C_{i} y_{i} \tilde{\hat{ y_{i}}} \prod _{j\in par(i)}y_{j}\hat{y_{j}} \end{aligned}$$

7 $$\begin{aligned} \ell _{2}&= w_{2}\sum ^{N-1}_{i=1} C_{i} y_{i} \tilde{\hat{ y_{i}}} \sum _{j\in par(i)}y_{j} \tilde{\hat{y_{j}}} \end{aligned}$$

8 $$\begin{aligned} \ell _{3}&= w_{3}\sum ^{N-1}_{i=1} C_{i} \tilde{y_{i}} \hat{ y_{i}} \prod _{j\in par(i)}y_{j} \hat{y_{j}} \end{aligned}$$

9 $$\begin{aligned} \ell _{4}&= w_{4}\sum ^{N-1}_{i=1} C_{i} \tilde{y_{i}} \hat{ y_{i}} \sum _{j\in par(i)} \tilde{y_{j}} \hat{y_{j}} \end{aligned}$$

$w_{1}$, $w_{2}$, $w_{3}$ and $w_{4}$ are weight constants that represent the weight of different errors in the loss function.

$C_i$ is the misclassification cost of a node *i*, and $C_i\ge 0$. The cost of misclassification of a positive instance as negative and the cost of misclassification of a negative instance as positive are considered, and the weights of such errors are expressed by $\alpha$ and $\beta$, respectively.

For a DAG hierarchy, as a node may have many parent nodes, $C_i$ is expressed as:

10 $$\begin{aligned} C_i=\left\{ \begin{array}{ll} 1, &{}i=0\\ \sum _{j\in par(i)}\frac{C_j}{\vert child(j)\vert }, &{}i>0 \end{array} \right. \end{aligned}$$

From the definition of the misclassification cost $C_i$, we can see that the misclassification cost $C_i$ is used to reflect the information of the hierarchical structure and to some extent reflects the importance of a node in the hierarchical structure. The closer a node is to the root node, the more important the node is, and the higher the cost of misclassification; conversely, the farther the node is from the root node, the less important the node is, and the lower the cost of misclassification.

Now we have finished the design of the hierarchical loss function, the DAGH loss function. Different from loss functions to be optimized in neural networks, our DAGH loss function is a principle used to compute hierarchical prediction error and construct the mathematical model later.

#### The BD mathematical model

By now, according to Bayesian decision theory, for an instance $\ {\mathbf {x}}$, the DAGH loss function is substituted into formula (2), and the form of conditional risk of $\ {\mathbf {x}}$ using the DAGH loss function can be obtained.

11 $$\begin{aligned} \begin{aligned} R(\hat{{\mathbf {y}}}\vert {\mathbf {x}})&=\sum _{{\mathbf {y}}\in \{0,1\}^N}L_{DAGH}({\mathbf {y}},\hat{{\mathbf {y}}})P({\mathbf {y}}\vert {\mathbf {x}})\\&=T_{1}+T_{2}+T_{3}+T_{4} \end{aligned} \end{aligned}$$

where,

12 $$\begin{aligned} T_{1}&= \sum _{{\mathbf {y}}}\left( w_{1}\sum ^{N-1}_{i=1} C_{i} y_{i} \tilde{\hat{ y_{i}}} \prod _{j\in par(i)}y_{j}\hat{y_{j}}\right) P({\mathbf {y}}\vert {\mathbf {x}}) \end{aligned}$$

13 $$\begin{aligned} T_{2}&= \sum _{{\mathbf {y}}}\left( w_{2}\sum ^{N-1}_{i=1} C_{i} y_{i} \tilde{\hat{ y_{i}}} \sum _{j\in par(i)}y_{j} \tilde{\hat{y_{j}}}\right) P({\mathbf {y}}\vert {\mathbf {x}}) \end{aligned}$$

14 $$\begin{aligned} T_{3}&= \sum _{{\mathbf {y}}}\left( w_{3}\sum ^{N-1}_{i=1} C_{i} \tilde{y_{i}} \hat{ y_{i}} \prod _{j\in par(i)}y_{j} \hat{y_{j}}\right) P({\mathbf {y}}\vert {\mathbf {x}}) \end{aligned}$$

15 $$\begin{aligned} T_{4}&= \sum _{{\mathbf {y}}}\left( w_{4}\sum ^{N-1}_{i=1} C_{i} \tilde{y_{i}} \hat{ y_{i}} \sum _{j\in par(i)} \tilde{y_{j}} \hat{y_{j}}\right) P({\mathbf {y}}\vert {\mathbf {x}}) \end{aligned}$$

For an instance ${\mathbf {x}}$, the probability of *i* at node $P(y_i=1\vert {\mathbf {x}})$ is abbreviated as $p_i$, that is, $p_i=P(y_i=1\vert {\mathbf {x}})$. $T_{1}$, $T_{2}$, $T_{3}$, and $T_{4}$ are expanded and collated, respectively, and the following proposition can be drawn.

##### **Proposition 1**

*In the DAG hierarchical* *multilabelclassification*, *a DAG hierarchy is defined as H*. *There are* *N* *nodes in* *H*, *which are denoted as* $\{0,1,2,\ldots ,N-1\}$, *and the* 0 *node is the root node. For an instance* ${\mathbf {x}}$, *the concrete form of conditional risk using* *the* *DAGH loss function is as follows*:

16 $$\begin{aligned} \begin{aligned} R(\hat{{\mathbf {y}}}\vert {\mathbf {x}})&=\sum _{{\mathbf {y}}\in \{0,1\}^N}L_{DAGH}({\mathbf {y}},\hat{{\mathbf {y}}})P({\mathbf {y}}\vert {\mathbf {x}})\\&=w_{1}\sum ^{N-1}_{i=1} C_{i}\tilde{\hat{ y_{i}}} p_{i} \prod _{j\in par(i)}\hat{y_{j}}\\&\quad + w_{2}\sum ^{N-1}_{i=1} C_{i} \tilde{\hat{ y_{i}}} p_{i} \sum _{j\in par(i)} \tilde{\hat{y_{j}}}\\&\quad +w_{3}\sum ^{N-1}_{i=1} C_{i} \hat{ y_{i}}\left( \prod _{j\in pa(i)}{p_{j}} -p_{i}\right) \prod _{j\in par(i)} \hat{y_{j}}\\&\quad +w_{4}\sum ^{N-1}_{i=1} C_{i} \hat{ y_{i}} \sum _{j\in par(i)}\hat{y_{j}}(1-p_{j}) \end{aligned} \end{aligned}$$

##### *Proof*

The unfolding process of $T_{1}$ is as follows:

17 $$\begin{aligned} \begin{aligned} T_{1}&=\sum _{{\mathbf {y}}}\left( w_{1}\sum ^{N-1}_{i=1} C_{i} y_{i} \tilde{\hat{ y_{i}}} \prod _{j\in par(i)}y_{j}\hat{y_{j}}\right) P({\mathbf {y}}\vert {\mathbf {x}})\\&=w_{1}\sum ^{N-1}_{i=1} C_{i}\tilde{\hat{ y_{i}}}p_{i}\prod _{j\in par(i)}\hat{y_{j}} \end{aligned} \end{aligned}$$

Similarly, the expansion process of $T_{2}$ is as follows:

18 $$\begin{aligned} \begin{aligned} T_{2}&=\sum _{{\mathbf {y}}}\left( w_{2}\sum ^{N-1}_{i=1} C_{i} y_{i} \tilde{\hat{ y_{i}}} \sum _{j\in par(i)}y_{j} \tilde{\hat{y_{j}}}\right) P({\mathbf {y}}\vert {\mathbf {x}})\\&=w_{2}\sum ^{N-1}_{i=1} C_{i}\tilde{\hat{ y_{i}}}p_{i}\sum _{j\in par(i)}\tilde{\hat{ y_{j}}} \end{aligned} \end{aligned}$$

For $$T_{3}$$:

19 $$\begin{aligned} T_{3}&= \sum _{{\mathbf {y}}}\left( w_{3}\sum ^{N-1}_{i=1} C_{i} \tilde{y_{i}} \hat{ y_{i}} \prod _{j\in par(i)}y_{j} \hat{y_{j}}\right) P({\mathbf {y}}\vert {\mathbf {x}})\nonumber \\&= w_{3}\sum ^{N-1}_{i=1} C_{i}\hat{ y_{i}} [P( {\mathbf {y}}_{par(i)}=1\vert {\mathbf {x}}) \nonumber \\&\quad-P(y_{i}=1, {\mathbf {y}}_{par(i)}=1\vert {\mathbf {x}})] \prod _{j\in par(i)}\hat{ y_{j}} \end{aligned}$$

20 $$\begin{aligned} \because P( {\mathbf {y}}_{par(i)}&= 1\vert {\mathbf {x}}) =\prod _{j\in par(i)}P( y_{j}=1\vert {\mathbf {x}})=\prod _{j\in par(i)}p_{j}\nonumber \\&\therefore T_{3}=w_{3}\sum ^{N-1}_{i=1} C_{i}\hat{ y_{i}}(\prod _{j\in par(i)}{p_{j}} -p_{i})\prod _{j\in par(i)} \hat{y_{j}} \end{aligned}$$

For $$T_{4}$$:

21 $$\begin{aligned} \begin{aligned} T_{4}&=\sum _{{\mathbf {y}}}\left( w_{4}\sum ^{N-1}_{i=1} C_{i} \tilde{y_{i}} \hat{ y_{i}} \sum _{j\in par(i)} \tilde{y_{j}} \hat{y_{j}}\right) P({\mathbf {y}}\vert {\mathbf {x}})\\&=w_{4}\sum ^{N-1}_{i=1} C_{i}\hat{ y_{i}}\sum _{j\in par(i)}\hat{ y_{j}}[P( y_j=0\vert {\mathbf {x}}) \\&\quad -P(y_{i}=1, y_j=0\vert {\mathbf {x}})]\\ \end{aligned} \end{aligned}$$

According to the hierarchical constraint, $P(y_{i}=1, y_{j}=0\vert {\mathbf {x}})=0$, therefore,

22 $$\begin{aligned} \begin{aligned} T_{4}&=w_{4}\sum ^{N-1}_{i=1} C_{i} \hat{ y_{i}}\sum _{j\in par(i)} \hat{y_{j}}(1-p_{j}) \end{aligned} \end{aligned}$$

Substituting $T_{1}$,$T_{2}$,$T_{3}$ and $T_{4}$ together, it is the exact equation proposed in Proposition 1. $\square$

According to Bayesian decision theory based on the principle of minimum risk, for a DAG hierarchical multilabel classification problem, its BD mathematical model can be written as:

23 $$\begin{aligned} \begin{aligned} \hat{{\mathbf {y}}^{*}}&=\arg \min _{\hat{{\mathbf {y}}}\in \Psi }R(\hat{{\mathbf {y}}}\vert {\mathbf {x}}) \\&=\arg \min _{\hat{{\mathbf {y}}}\in \Psi }\sum _{{\mathbf {y}}\in \{0,1\}^N}L_{DAGH}({\mathbf {y}},\hat{{\mathbf {y}}})P({\mathbf {y}}\vert {\mathbf {x}}) \end{aligned} \end{aligned}$$

Although the BD mathematical model has been built, the formula (23) is difficult to solve. The simplification of this model is described in detail in the following subsection.

#### The simplification of the BD mathematical model

A solution to simplify the BD model is derived by further deducing and sorting out formula (23), which transforms the minimization problem described by formula (23) into the maximization problem described by Proposition 2. The specific contents of this proposition are as follows.

##### **Proposition 2**

*The problem of minimizing the risk function expressed in formula* (23) *is equivalent to the following optimization problem*:

24 $$\begin{aligned} \hat{{\mathbf {y}}}^{*}=\arg \max _{\hat{{\mathbf {y}}}\in \Psi } LE_{\delta }(\hat{{\mathbf {y}}},{\mathbf {x}}) \end{aligned}$$

where $LE_{\delta }(\hat{{\mathbf {y}}},{\mathbf {x}})$ *is defined as*:

25 $$\begin{aligned} \begin{aligned} LE_{\delta }(\hat{{\mathbf {y}}},{\mathbf {x}})&=w_{2}\sum ^{N-1}_{i=1}C_{i} p_{i}\sum _{j\in par(i)}\hat{y_{j}} \\&\quad -w_{1}\sum ^{N-1}_{i=1}C_{i} p_{i}\prod _{j\in par(i)}\hat{y_{j}}\\&\quad +\sum ^{N-1}_{i=1}\hat{ y_{i}}[w_{1}C_{i} p_{i} -w_{3}C_{i}\left( \prod _{j\in par(i)}{p_{j}} -p_{i}\right) \\&\quad -w_{4}C_{i}\sum _{j\in par(i)}(1-p_{j})] \end{aligned} \end{aligned}$$

##### *Proof*

The items $T_{1}$,$T_{2}$,$T_{3}$, and$T_{4}$ expressed in formulas (17), (18), (20), and(21) are further sorted. The expansion of $T_{1}$ and $T_{2}$ are as follows:

26 $$\begin{aligned} T_{1}&= w_{1}\sum ^{N-1}_{i=1} C_{i}\tilde{\hat{ y_{i}}}p_{i}\prod _{j\in par(i)}\hat{y_{j}}\nonumber \\&= w_{1}\sum ^{N-1}_{i=1} C_{i}p_{i}\prod _{j\in par(i)}\hat{y_{j}} -w_{1}\sum ^{N-1}_{i=1}C_{i}p_{i}\hat{ y_{i}} \end{aligned}$$

27 $$\begin{aligned} T_{2}&= w_{2}\sum ^{N-1}_{i=1} C_{i}\tilde{\hat{ y_{i}}}p_{i}\sum _{j\in par(i)}\tilde{\hat{ y_{j}}}\nonumber \\&= w_{2}\sum ^{N-1}_{i=1} C_{i}p_{i}|par(i)|-w_{2}\sum ^{N-1}_{i=1} c_{i}p_{i}\sum _{j\in par(i)}\hat{ y_{j}} \end{aligned}$$

Similarly, for $T_{3}$ and $T_{4}$:

28 $$\begin{aligned} T_{3}&= w_{3}\sum ^{N-1}_{i=1} C_{i} \hat{ y_{i}}\left( \prod _{j\in par(i)}{p_{j}} -p_{i}\right) \prod _{j\in par(i)} \hat{y_{j}}\nonumber \\&= w_{3}\sum ^{N-1}_{i=1} C_{i} \hat{ y_{i}}\left( \prod _{j\in par(i)}{p_{j}} -p_{i}\right) \end{aligned}$$

29 $$\begin{aligned} T_{4}&= w_{4}\sum ^{N-1}_{i=1} C_{i} \hat{ y_{i}}\sum _{j\in par(i)} \hat{y_{j}}(1-p_{j})\nonumber \\&= w_{4}\sum ^{N-1}_{i=1} C_{i} \hat{ y_{i}}\sum _{j\in par(i)} (1-p_{j}) \end{aligned}$$

By adding up the results of the above four items, we can obtain the following results:

30 $$\begin{aligned} \begin{aligned} R(\hat{{\mathbf {y}}}\vert {\mathbf {x}})&=w_{2}\sum ^{N-1}_{i=1}C_{i} p_{i}\vert par(i)\vert \\&\quad -\left\{ w_{2}\sum ^{N-1}_{i=1}C_{i} p_{i}\sum _{j\in par(i)}\hat{y_{j}}\right. \\&\left. \quad -w_{1}\sum ^{N-1}_{i=1}C_{i} p_{i}\prod _{j\in par(i)}\hat{y_{j}}\right. \\&\left. \quad +\sum ^{N-1}_{i=1}\hat{ y_{i}}\left[ w_{1}C_{i} p_{i} -w_{3}C_{i}\left( \prod _{j\in par(i)}{p_{j}} -p_{i}\right) \right. \right. \\&\left. \left. \quad -w_{4}C_{i}\sum _{j\in par(i)}(1-p_{j})\right] \right\} \end{aligned} \end{aligned}$$

Therefore, $L_{DAGH}(\hat{{\mathbf {y}}},{\mathbf {x}})$ can be written as:

31 $$\begin{aligned} R(\hat{{\mathbf {y}}}\vert {\mathbf {x}}) =w_{2}\sum ^{N-1}_{i=1}C_{i} p_{i}\vert par(i)\vert -LE_{\delta }(\hat{{\mathbf {y}}},{\mathbf {x}}) \end{aligned}$$

It is easy to see that the first item of the formula is independent of $\hat{{\mathbf {y}}}$, so the problem of finding the minimum value of $L_{DAGH}(\hat{{\mathbf {y}}},{\mathbf {x}})$ is to find the maximum value of $LE_{\delta }(\hat{{\mathbf {y}}},{\mathbf {x}})$, so the proof ends.

Because there are still two variables ${\hat{y}}_i$ and $\hat{{\mathbf {y}}}_{par(i)}$ in the formula (25), it is difficult to solve the maximum value problem of $LE_{\delta }(\hat{{\mathbf {y}}},{\mathbf {x}})$. To solve this problem, a node function $\sigma (\cdot )$ is introduced. A node *i* is specifically defined as:

32 $$\begin{aligned} \sigma (i)=\left\{ \begin{array}{ll} \sigma _{1}(i) , &{}i=0\\ \sigma _{1}(i)+\sigma _{2}(i), &{}i>0 \end{array} \right. \end{aligned}$$

where, $\sigma _{1}(i)$ and $\sigma _{2}(i)$ are defined as:

33 $$\begin{aligned} \sigma _{1}(i)&= \sum _{j\in child(i)}w_{2}C_{j} p_{j}-\prod _{j\in child(i)}w_{1}C_{j} p_{j} \end{aligned}$$

34 $$\begin{aligned} \sigma _{2}(i)&= w_{1}C_{i} p_{i} -w_{3}C_{i}\left( \prod _{j\in par(i)}{p_{j}} -p_{i}\right) \nonumber \\&\quad-w_{4}C_{i}\sum _{j\in par(i)}(1- p_{j}) \end{aligned}$$

In particular, when the children node set of node *i* is an empty set, the value of $\sigma _1(i)$ is 0, that is, when $child(i)=\varnothing$, $\sigma _1(i)=0$. The definition of the function $\sigma _2(i)$ does not include the root node.

After introducing the concept of node function $\sigma (i)$, $LE_{\delta }(\hat{{\mathbf {y}}},{\mathbf {x}})$ can be represented by $\sigma (i)$. $\square$

##### **Proposition 3**

$LE_{\delta }(\hat{{\mathbf {y}}},{\mathbf {x}})$ *is expressed by* $\sigma (i)$ *as*:

35 $$\begin{aligned} LE_{\delta }(\hat{{\mathbf {y}}},{\mathbf {x}})=\sum _{i}\hat{y_{i}}\sigma (i) \end{aligned}$$

##### *Proof*

Let $M=\sum _{i}\hat{y_{i}}\sigma (i)$, and unfolding it, we can obtain:

36 $$\begin{aligned} \begin{aligned} M&=\sum _{i}\hat{y_{i}}\sigma (i)\\&=\sum _{i>0}\hat{y_{i}}[\sigma _{1}(i)+\sigma _{2}(i)]+\hat{y_{0}}\sigma _{1}(0)\\&=\sum _{i}\hat{y_{i}}\sigma _{1}(i)+\sum _{i>0}\hat{y_{i}}\sigma _{2}(i) \end{aligned} \end{aligned}$$

Let the first item of *M* be $M_{1}$, so $M_{1}=\sum _{i}\hat{y_{i}}\sigma _{1}(i)$, and unfolding $M_{1}$:

37 $$\begin{aligned} \begin{aligned} M_{1}&=\sum _{i}\hat{y_{i}}\sigma _{1}(i)\\&=\sum _{i}\hat{y_{i}}[\sum _{j\in child(i)}w_{2}C_{j} p_{j}-\prod _{j\in child(i)}w_{1}C_{j} p_{j}]\\&=w_{2}\sum _{j>0}C_{j} p_{j}\sum _{k\in par(j)}{\hat{y}}_{k} -w_{1}\sum _{j>0}C_{j} p_{j}\prod _{k\in par(j)}{\hat{y}}_{k} \end{aligned} \end{aligned}$$

Let the second item of *M* be $M_{2}$, so $M_{2}=\sum _{i>0}\hat{y_{i}}\sigma _{2}(i))$, and unfolding $M_{2}$:

38 $$\begin{aligned} \begin{aligned} M_{2}&=\sum _{i>0}\hat{y_{i}}\sigma _{2}(i))\\&=\sum _{i>0}\hat{y_{i}}[w_{1}C_{i} p_{i} -w_{3}C_{i}\left( \prod _{j\in par(i)}{p_{j}} -p_{i}\right) \\&\quad -w_{4}C_{i}\sum _{j\in par(i)}(1- p_{j})] \end{aligned} \end{aligned}$$

$$\square$$

Combining both $M_{1}$ and $M_{2}$, $M_{1}+M_{2}=LE_{\delta }(\hat{{\mathbf {y}}},{\mathbf {x}})$ is proved.

Based on Proposition 3, the solution to the problem described in Proposition 2 is equivalent to the following problem.

##### **Proposition 4**

*For a DAG hierarchical* *multilabel* *classification problem, when* *the* *DAGH loss function is used, according to Bayesian decision theory, the classification can be transformed into the following optimization problems*.

39 $$\begin{aligned} \hat{{\mathbf {y}}}^{*}=\arg \max _{\hat{{\mathbf {y}}}\in \Psi } \sum _{i}{\hat{y}}_{i}\sigma (i) \end{aligned}$$

*Formula* (39) *is the simplified mathematical model for* *the DAG hierarchical multilabel* *classification problem*. *To solve the optimization problem described by formula* (39), *only the posterior probability of an instance at each node needs to be obtained*, *which* *is exactly what we described in the earlier section* *when discussing* *the predicted probability for each label through the LSTM network*. *According to the strategy described in formula* (39), *we can* *obtainthe optimized result of the label set of each instance*.

### Hierarchical constraint algorithm DAGLabel

In solving the optimization problem above, it is necessary to ensure that the final classification result $\hat{{\mathbf {y}}}^{*}$ satisfies the DAG hierarchical constraint, i.e., conforms to formula (1). To ensure that the final result satisfies the hierarchical constraint, the DAGLabel algorithm is used to solve the optimization problem in formula (39).

DAGLabel is a greedy algorithm. Its basic idea is to traverse the whole hierarchical structure and select the node with the largest $\sigma$ value for judgment and operation. The DAGLabel algorithm simplifies the DAG step by step and finds the optimal classification result by searching for the node with maximum $\sigma$ in the hierarchical structure and combining it with the parent node, which may violate the hierarchical constraint as a new node. The DAGLabel algorithm can obtain the optimal classification result without knowing the maximum number of labels for an unknown instance and can ensure that the classification result meets the hierarchical constraint. The pseudocode of the DAGLabel algorithm is described in algorithm 1. 
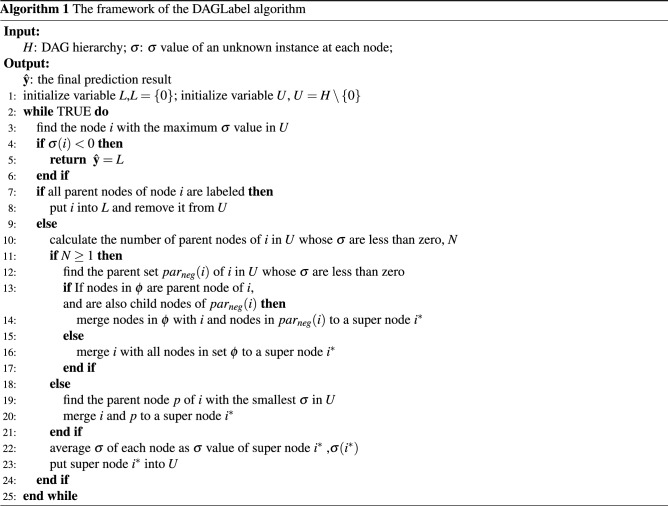

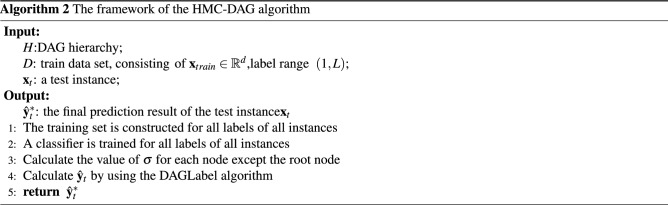


### The main steps of the HLSTMBD algorithm

The proposed mathematical model transforms the DAG hierarchical multilabel classification problem into classical classification problems, which provides a theoretical basis for the algorithm design of DAG hierarchical multilabel classification problems. This section combines the three parts above and shows how the entire model works properly.

The hierarchical multilabel classification algorithm proposed in this paper is abbreviated as the HMC-DAG algorithm. This algorithm consists of two parts: a training stage and a prediction stage. In the training stage, a single classifier is trained in the directed acyclic graph. In the prediction stage, for an unknown instance ${\mathbf {x}}$, the algorithm mainly includes the following three steps.

*Step 1* Use the base classifier obtained in the training stage to classify instance ${\mathbf {x}}$ separately, and obtain the classification result.

*Step 2* Calculate the value of $\sigma$ at each node by using the results of step 1 and the formulas (32), (33) and (34).

*Step 3* Use the value of $\sigma$ obtained in step 2 and formula (39) to calculate the final classification result $\hat{{\mathbf {y}}}^{*}$.

The overall framework of the DAG hierarchical multilabel classification algorithm proposed in this paper is given in algorithm 2.

#### Complexity analysis

In this section, the complexity of the HMC-DAG algorithm is analysed. This algorithm designs a classifier in the training stage and transforms the hierarchical multilabel classification problem into a ’seq-seq’ classification problem for processing, so the complexity of step 1 depends on the selected base classifier.

Step 2 calculates the value $\sigma$ of an instance at each node. If there are *N* nodes in the DAG, for an instance to be tested, the time complexity is *O*(*N*).

Step 3 calculates the final classification result, which is implemented by the DAGLabel algorithm, so the time complexity of this part depends on the DAGLabel algorithm. After the analysis of the DAGLabel algorithm, the time complexity of this part is $O(N\log (N))$.

## Experiment

The main application area of DAG hierarchical multilabel classification is biological function prediction. To validate the performance of the hierarchical multilabel classification algorithm proposed in this paper, we designed an experiment using data sets in the field of lncRNA function prediction, and selected Gene Ontology as the gene function annotation scheme. To design related experiments, we should first analyze the specific content of the annotation scheme of gene function used in the experiment.

### GO annotation scheme

With the development of biology, the biological function annotation scheme using tree structure is too simple to organize and describe the complex relationship between biological functions. Therefore, Gene Ontology (GO) has been widely used by researchers^28^. The GO annotation scheme can effectively organize complex information between biological functions and has become a comparatively popular biological function annotation scheme^29^.

The GO annotation scheme was proposed by the Gene Ontology Consortium to describe and manage the functions of genes and their products in various species. It annotates genes or proteins by using proprietary biological terms^30^. GO can be represented by a directed acyclic graph, in which each node corresponds to a function and each directed edge corresponds to the membership relationship between nodes. Figure 1 is a part of GO.

### Experimental datasets

The dataset GOA-lncRNA used in the experiment comes from biological lncRNA data under GO annotation. Currently, scientists usually infer functions of lncRNAs by the interactions between DNA,RNA,proteins and them^12^. These interactions may contribute to cellular processes. Since genes with the same or similar functions often have similar expression patterns in multiple different tissues, it is an effective method to analyse the role of lncRNAs by analysing the coexpression patterns shared with neighbouring counterparts.

Since there is currently no public GO annotation for lncRNAs, but based on the fact that the target lncRNA may have a very similar function to the direct neighbour protein in the lncRNA protein binding network, the literature uses the known protein GO annotation to annotate some lncRNAs using the neighborhood counting method. That is, for each target lncRNA l in the lncRNA-protein association network, the occurrence frequency of each function f belonging to F is calculated based on the direct neighbours of l, where F is a set of functions owned by all direct neighbors of l. If it has the f function, it will be labelled 1; otherwise, it will be labelled 0. At the same time, an appropriate neighbour threshold as analysed in Zhang’s work^1^ is selected to adjust the prediction of lncRNAs; thereby, the initial dataset of this article, GOA-lncRNAs, is obtained. After selecting the biological process aspect of GO terms as labels, the dataset with features and labels is randomly divided as $D_{1}$–$D_{5}$.

The specific content of each data set is shown in Table 1.

**Table 1:** Information from 5 datasets.

Dataset	Instances number|*D*|	Attribute number |*A*|
$D_{1}$	807	50
$D_{2}$	807	50
$D_{3}$	807	50
$D_{4}$	807	50
$D_{5}$	808	50

### Evaluation criteria

Although no evaluation method is considered to be the best evaluation criterion for the DAG hierarchical multi-label classification problem at present, micro average $F_1$ and macro average $F_1$ are recommended as evaluation criteria of the hierarchical multi-label classification problem in paper^31^. Both are suitable for evaluating DAG hierarchical multilabel classification. These two indicators are widely used in many studies,such as^32–35^.

Let a data set contain a total of *m* instances. The precision $hPre^{\mu }$, recall $hRec^{\mu }$ and $F_1$ indicator $hF_{1}^{\mu }$ in the form of microaverage are calculated as follows.

40 $$\begin{aligned} hPre^{\mu }&= \frac{\sum _{i=1}^{m} \vert \hat{P_{i}} \bigcap \hat{T_{i}}\vert }{\sum _{i=1}^{m}\vert \hat{P_{i}}\vert } \end{aligned}$$

41 $$\begin{aligned} hRec^{\mu }&= \frac{\sum _{i=1}^{m} \vert \hat{P_{i}} \bigcap \hat{T_{i}}\vert }{\sum _{i=1}^{m}\vert \hat{T_{i}}\vert } \end{aligned}$$

42 $$\begin{aligned} hF_{1}^{\mu }&= \frac{2\times hPre^{\mu }\times hRec^{\mu }}{hPre^{\mu }+hRec^{\mu }} \end{aligned}$$

The macroaverage version of precision $hPre^{M}$, recall $hRec^{M}$ and the $F_1$ value $hF_{1}^{M}$ are calculated as follows.

43 $$\begin{aligned} hPre^{M}&=\frac{\sum _{i=1}^{m} hPre_{i}}{m} \end{aligned}$$

44 $$\begin{aligned} hRec^{M}&=\frac{\sum _{i=1}^{m} hRec_{i}}{m} \end{aligned}$$

45 $$\begin{aligned} hF_{1}^{M}&=\frac{\sum _{i=1}^{m} hF_{1,i}}{m} \end{aligned}$$

For the $i-th$ instance in the dataset, the definition of its hierarchical precision ($hPre_{i}$),hierarchical recall ($hRec_{i}$) and hierarchical $F_1$($hF_{1,i}$)are:

46 $$\begin{aligned} hPre_i&= \frac{\vert \hat{P_{i}} \bigcap \hat{T_{i}}\vert }{\vert \hat{P_{i}}\vert } \end{aligned}$$

47 $$\begin{aligned} hRec_i&= \frac{\vert \hat{P_{i}} \bigcap \hat{T_{i}}\vert }{\vert \hat{T_{i}}\vert } \end{aligned}$$

48 $$\begin{aligned} hF_{1,i}&= \frac{2\times hPre_i\times hRec_i}{hPre_i+hRec_i} \end{aligned}$$

Also, $\hat{P_{i}}$ is the set consisting of the most specific class(es) predicted for a test instance and all its(their) ancestor classes, and $\hat{T_{i}}$ is the set consisting of the most specific true class(es) of test example i and all its(their) ancestor classes. We can see that the micro average $hF_{1}^{\mu }$ is calculated by precision and recall on all the samples; however, the macro average $hF_{1}^{M}$ is calculated by $hPre_{i}$ and $hRec_{i}$ for each sample. It should be noted that there is a common characteristic for all metrics; the larger the measure value is, the better the classifier performance.

### Experimental setup

In terms of hardware equipment, the computer CPU used in this experiment has eight cores and sixteen threads, the main frequency is 2.9 GHz, NVIDIA (R) Cuda version 11.1, and the multicore in the graphics card can be used for calculation and processing to improve calculation performance. The biological process aspect of GO is our focus in the experiment section.As previously described in papers^36,37^, we use two-thirds of each data set for training and the remaining one-third for testing. Out of the training set, two-thirds are used for the actual training, and one-third is used to validate the parameters.

The classifier model used in the experiment is built through the Keras API, which uses Tensorflow as the backend. The advantage of Keras is that it sets the commonly used neural network layer into a modular API, which is convenient for building compilation and calling. The model can classify all labels at once. The learning rate of 0.0008 is automatically selected and returned through “RandomizedSearchCV” from a list of learning rates. Activation in all dense layers is selected as “sigmoid”,while in the LSTM layer, it is selected as “tanh” by default. The neural network is trained by optimizing the binary crossentropy error.

The SVM classification in the comparative experiment is realized through the LIBSVM package^38^. LIBSVM is a software package developed by Professor Lin Zhiren of National Taiwan University to implement the SVM algorithm. The author provides many default parameters for the SVM, which simplifies the process of using and adjusting the parameters of the SVM. We choose RBF (radial basis function) as the kernel function in the experiment. The classification follows the following steps: convert the original data to the SVM format, perform the data normalization, use the radial basis kernel function, use cross-validation to find the optimal parameters C and $\gamma$, and finally use the optimal parameters to train the entire datasets and test.

### Results and analysis

#### The results of the HMC-DAG algorithm

To verify the effectiveness and performance of the proposed HMC-DAG algorithm, the algorithm is used to deal with lncRNA function prediction based on the GO scheme for the 5 datasets described above, and the results are evaluated by using both the macroaveraged version and the microaveraged version of $hF_1$. Table 2 show the results of HMC-DAG when using LSTM as the base classifier.

**Table 2:** The results of HMC-DAG-LSTM.

Data set	HMC-DAG-LSTM	
	$hF_{1}^{\mu }$	$hF_{1}^{M}$
D1	0.940	0.933
D2	0.927	0.917
D3	0.958	0.958
D4	0.933	0.926
D5	0.960	0.960

#### Compared with other algorithms

To illustrate the superiority of our proposed HLSTMBD method, we combine this algorithm with eight other baseline algorithms, HMC-TD-SVM, HMC-DT-SVM, HMC-TPR-SVM, HMC-DAG-SVM, HMC-TD-LSTM, HMC-DT-LSTM, HMC-TPR-LSTM, and CLUS-HMC, for comparison.

The comparison algorithm can be divided into two categories. The CLUS-HMC algorithm is a global method. The method sets different weights for different labels in the hierarchical structure, generates an inductive decision tree to classify all labels at once, and uses the weighted Euclidean distance as a measure and cross-validation to determine the required level parameters^39^. FTest is the stopping criterion, and the node will be split only when the internal variance of FTest in a certain level of subset is significantly reduced. The CLUS-HMC method can establish a set of FTests and optimize them. In this case, the smallest FTest will be selected to minimize the RMSE metric on the provided validation set.

The remaining methods are local methods. First, a base classifier is set to initially classify the labels. Since the label results obtained by the base classifier may violate the given hierarchical relationship, the hierarchical constraint algorithm is added to modify the classification results to make the classification results effective. The comparison method is compared with SVM at the base classifier level and compared with the TOPDOWN, DOWNTOP, and TPR algorithms at the hierarchical constraint algorithm level.

The SVM classifier aims to find an optimal hyperplane that can distinguish between various types, so that the distance between the parallel and the optimal hyperplane and the support vector is maximized, and each sample point is mapped to an infinite-dimensional feature space through the kernel function. The dimension ascends, so that the originally linearly inseparable space becomes linearly separable. The SVM classifier has been proven to have excellent generalization ability and can effectively avoid overfitting problems, and since the solving process is a convex optimization problem, its local optimal solution must be the global optimal solution.

The comparison method, the TPR method is called the true path rule and is a common method to integrate the initial classification results^40^. The whole method process can be divided into two steps, from the bottom to the top (downtop step) and from the top to the bottom (topdown step). To traverse the entire hierarchical structure from bottom to top, pass the positive prediction value of the lower node to the upper node at first, so that it has an impact on the judgment of the upper node. After this process is over, the hierarchical structure is accessed from the root node from top to bottom, and the result of the upper-level node whose prediction result is still negative is passed to the relevant lower-level node. The downtop and topdown steps in the TPR method represent the bottom-up and top-down steps, respectively, which can be used as the hierarchical constraint downtop and topdown methods to integrate the results separately.

**Figure 4:**
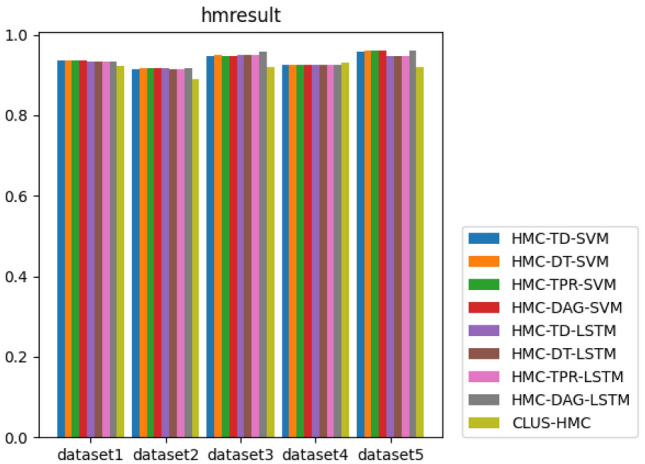
The bar chart of each algorithm’s $hF_{1}^{M}$ value.

**Figure 5:**
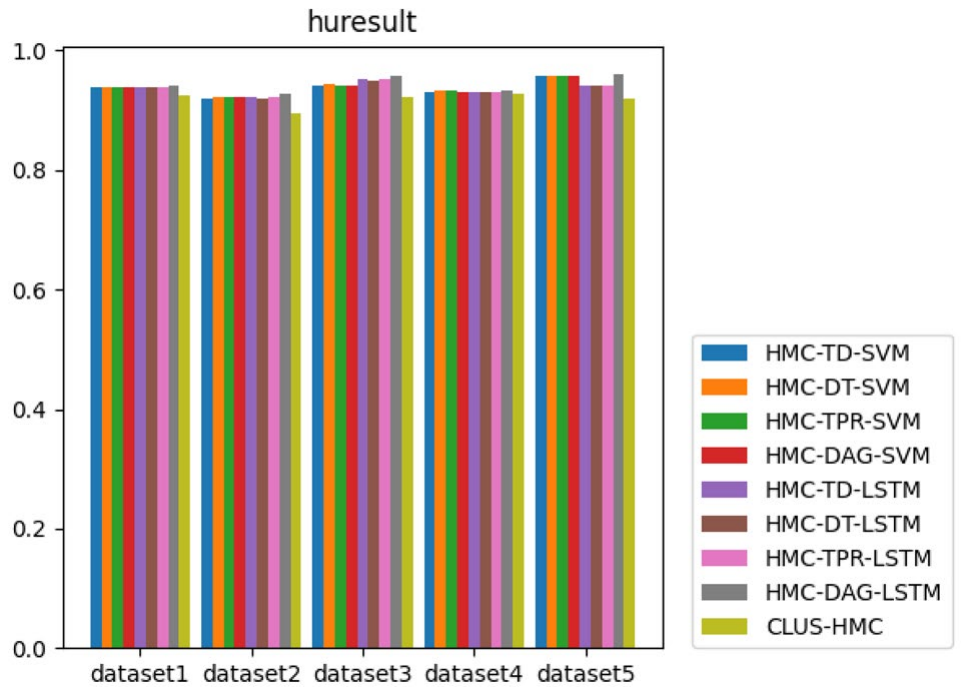
The bar chart of each algorithm’s $hF_{1}^{\mu }$ value.

The algorithm compares the two indicators of the microaveraged version and the macroaveraged version of $hF_1$, and the results are shown in Figs. 4 and 5. Of the ten indicators on the five datasets, eight of the HMC-DAG-LSTM algorithms are in a close or leading position. In addition, since the SVM in the experiment sets a classifier for each node to perform independent binary classification, each sample needs to integrate the binary prediction results of all labels, and LSTM classification only designs a classifier to complete the conversion from “sequence-sequence”, which can achieve the prediction results of all labels at one time in the output level. Therefore, the algorithm simplifies the classification and integration steps from the original design of a classifier for each node to a one-time classification for all nodes.

**Figure 6:**
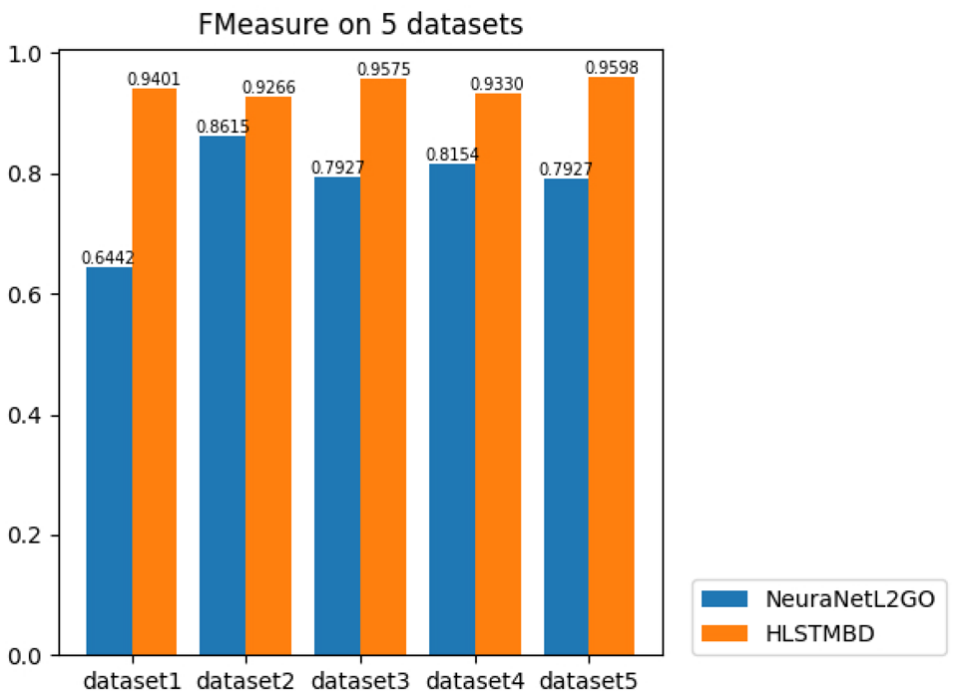
Bar chart of *Fmeasure* values of the two algorithms.

To fully certify the advantage of our approach, two more experiments were carried out to compare with the SOTA machine learning method, the NeuraNetL2GO method, and gave a possible explanation for why our method showed better results. A classifier chain in accord with the hierarchy structure formed by many classifiers is trained in NeuraNetL2GO. The input of classifiers (except the classifier for the first level) consists of two parts: the outputs of the former classifier and the input feature vectors of the instances. In this way, the prediction of levels close to the root can have an impact on levels away from the root. First, we tested how the NeuraNetL2GO method worked on our 5 datasets. Since the definition of the evaluation metric Fmax in NeuraNetL2GO is actually the evaluation metric result in our approach, the results are shown below in Fig. 6. According to our GO depth, the percentage of hidden units is set to [0.6,0.55,0.5,0.45,0.4,0.35,0.3,0.25,0.2,0.15,0.1].

**Figure 7:**
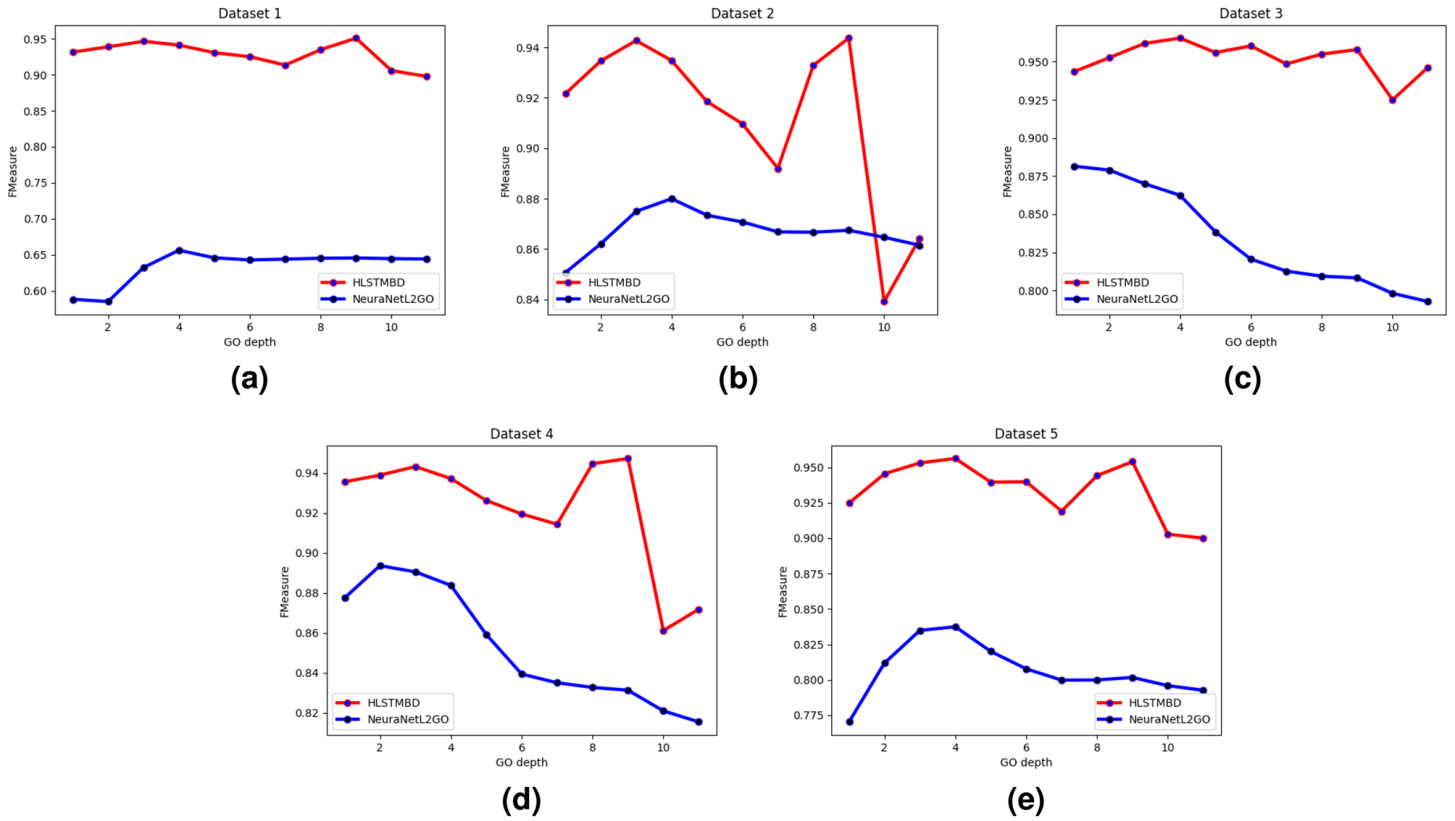
Performance comparison of different levels in the GO hierarchy.

The results in Fig. 6 show that of all 5 datasets, our method has a higher F-measure, which indicates a better prediction model. To explain the results in more depth, Fig. 7 shows the Fmeasure-GO depth on 5 datasets. It can be seen that our methods performed better in nearly all levels of prediction, and these figures indicate that as the levels go deeper, the results of the NeuraNetL2GO method show an obvious descent tendency at approximately level 4, while the broken lines in our method are not all the way down. Different from the classifier chain in which the performance of the former classifiers has a direct impact on the latter ones, only one classifier is used to complete our classification, which means that one GO term in the GO sequence has relatively more independence on its own prediction result determined by its own input features and has little influence on others’ prediction.

**Figure 8:**
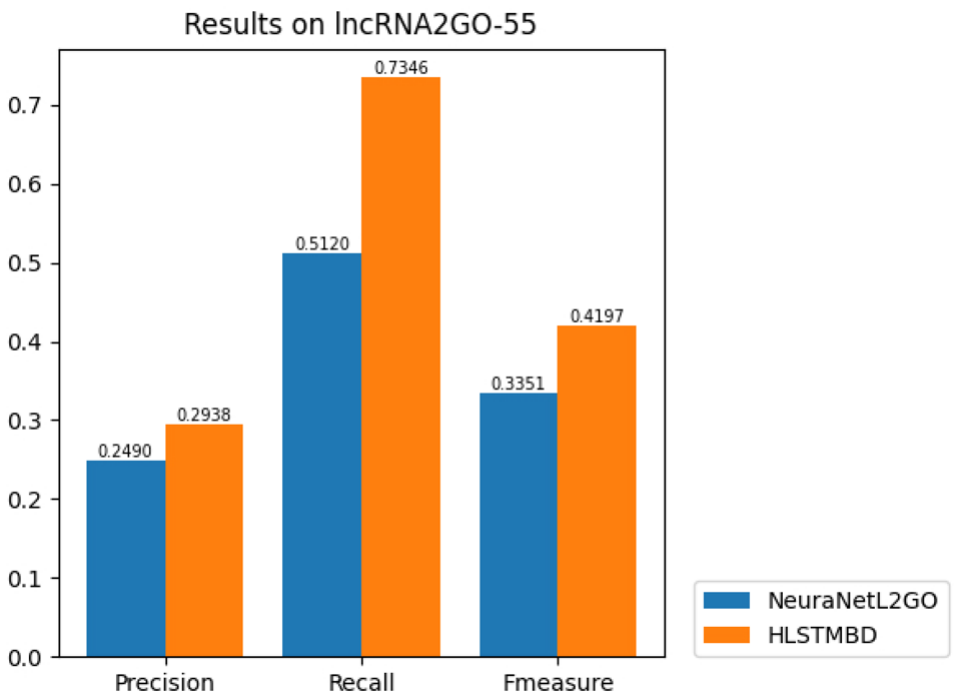
The precision, recall, and Fmeasure of the two methods.

**Figure 9:**
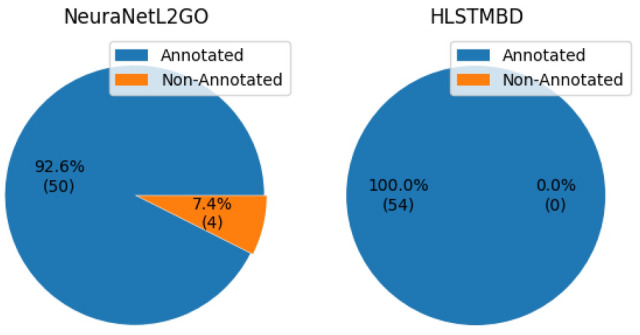
The numbers of lncRNAs that are annotated correctly by the two methods, respectively.

In another experiment, our method is applied to the lncRNA2GO-55 dataset, the test dataset of the NeuraNetL2GO method manually created based on Zhang’s work, which only includes lncRNAs that have been functionally characterized through knockdown or overexpression experiments. The results in Fig. 8. show that our HLSTMBD method has a higher Fmeasure of 8.46$\%$ than the NeuraNetL2GO method. We further calculated the numbers of lncRNAs that were annotated with at least one biological process GO term, and the results are shown below in Fig. 9. All 54 lncRNAs were annotated with at least one GO term in our method; however, the number in NeuraNetL2GO was 50.

The effectiveness of our method is mainly due to two reasons. First, the advanced mathematical HBD model behind our method is a more precise and convincing theory for hierarchical multilabel classification problems. Different from NeuraNetL2GO, in which only the probability of nodes is considered and adjusted, our method is more comprehensive because the impact of the hierarchy structure is also involved. The framework also showed great generality, and there are many solutions to the classical classification problem simplified by this model. Next, the bidirectional LSTM network we use is well known for its excellent performance in capturing hidden global information, which indicates that the final result of the descendant nodes may have an effect on the ancestor nodes, and even if the two nodes are far apart, the latent relationship may also be noticed in our network. Compared to the classifier chain formed by MLPs used in NeuraNetL2GO, the hierarchy information is spread level by level in one direction and may have a great reduction as the level goes deeper; in other words, the node of the 2nd level may have little impact on the 10th level, and the chain may lead to the result that the prediction errors are easier to propagate between the levels. In addition, we use a single classifier. Compared to the classifier chain formed by the classifiers whose numbers are related to the scale of the hierarchy, our method is more flexible and has a smaller size.

In addition, we mainly focused on the BP (biological process) terms (one of the three groups of GO) of GO annotations, while the others(molecular function and cellular component) were also included in the NeuraNetL2GO method. In the area of the prediction of BP terms (an independent DAG structure), our method has better performance. In regard to the demand of predicting all GO groups (three separate DAG structures) at one time, three separate networks will be constructed in our method, and the current NeuraNetL2GO may be the better choice.

However, there may be some limitations in our HLSTMBD method. First, because of the lack of annotated lncRNA functions, a ’guilty by association’ method is applied to annotate lncRNAs; however, the annotations we obtain and send to classifiers may lead to a bias against the correct annotation. Next, although our BD model is proven to have great potential in hierarchical multilabel classification problems, the advantage of our constraint algorithm DAGLabel has not been fully revealed in the experiment against the TOPDOWN, DOWNTOP and TPR algorithms.

## Conclusion

In this paper, a new hierarchical multilabel classification method based on LSTM and Bayesian decision theory is proposed for lncRNA function prediction. First, the LSTM network is designed to capture part of the hierarchical relationship to complete the preliminary classification. Furthermore,a mathematical classification model based on the Bayesian decision theory (BD model) is constructed to fully consider and make use of the hierarchical information between parent and child nodes in the DAG structure, which can change the hierarchical multilabel classification problem to the conditional risk minimization problem. Finally, the hierarchical constraint algorithm DAGLabel is used to solve the BD model to obtain the final results, which can also correct the labels that violate the hierarchy constraints in the initial classification results. Experiments on five datasets show that compared to other baseline algorithms, the proposed HLSTMBM algorithm can classify all labels at one time without losing the precision in the classification stage, which greatly improves the classification efficiency and plays an important role in lncRNA function annotation.

In the future, a new prediction model may be built with fewer parameters or less labelled data to reduce the prediction time or improve the prediction performance.

## References

[1] Zhang J, Deng L (2018). Gene ontology-based function prediction of long non-coding rnas using bi-random walk. BMC Med. Genomics.

[2] Zhang Z, Zhang J, Fan C, Tang Y, Deng L (2017). Katzlgo: Large-scale prediction of lncrna functions by using the katz measure based on multiple networks. IEEE/ACM Trans. Comput. Biol. Bioinform..

[3] Dong H (2018). Exosome-mediated transfer of lncrna-snhg14 promotes trastuzumab chemoresistance in breast cancer. Int. J. Oncol..

[4] Liu HY (2020). lncrna slc16a1-as1 as a novel prognostic biomarker in non-small cell lung cancer. J. Investig. Med..

[5] Zhang, Y. *et al.* Overexpression of lncrna bm466146 predicts better prognosis of breast cancer. *Front. Oncol.* 3211 (2021). 10.3389/fonc.2020.628757 PMC7878538 33585256

[6] Muppirala UK, Honavar VG, Dobbs D (2011). Predicting rna-protein interactions using only sequence information. BMC Bioinform..

[7] Chen X (2019). Computational models for lncrna function prediction and functional similarity calculation. Brief. Funct. Genomics.

[8] Radivojac P (2013). A large-scale evaluation of computational protein function prediction. Nat. Methods.

[9] Guo X (2013). Long non-coding rnas function annotation: A global prediction method based on bi-colored networks. Nucleic Acids Res..

[10] Zhao J, Ma X (2019). Multiple partial regularized nonnegative matrix factorization for predicting ontological functions of lncrnas. Front. Genet..

[11] Jiang, Q. *et al.* Lncrna2function: A comprehensive resource for functional investigation of human lncrnas based on rna-seq data. In *BMC Genomics*, vol. 16, 1–11 (BioMed Central, 2015). 10.1186/1471-2164-16-S3-S2 PMC4331805 25707511

[12] Zhang J, Zhang Z, Wang Z, Liu Y, Deng L (2018). Ontological function annotation of long non-coding rnas through hierarchical multi-label classification. Bioinformatics.

[13] Yang C (2018). Lncadeep: An ab initio lncrna identification and functional annotation tool based on deep learning. Bioinformatics.

[14] Consortium GO (2019). The gene ontology resource: 20 years and still going strong. Nucleic Acids Res..

[15] The gene ontology resource. enriching a gold mine. *Nucleic Acids Res.* **49**, D325–D334 (2021). 10.1093/nar/gkaa1113 PMC7779012 33290552

[16] Feng S, Fu P, Zheng W (2017). A hierarchical multi-label classification algorithm for gene function prediction. Algorithms.

[17] Tennant PW (2021). Use of directed acyclic graphs (dags) to identify confounders in applied health research: Review and recommendations. Int. J. Epidemiol..

[18] Feng S, Zhao C, Fu P (2020). A deep neural network based hierarchical multi-label classification method. Rev. Sci. Instrum..

[19] Daisey K, Brown SD (2020). Effects of the hierarchy in hierarchical, multi-label classification. Chemom. Intell. Lab. Syst..

[20] Zhang L, Shah SK, Kakadiaris IA (2017). Hierarchical multi-label classification using fully associative ensemble learning. Pattern Recogn..

[21] Valentini, G. Notes on hierarchical ensemble methods for dag-structured taxonomies *Comput. Sci.* (2014).

[22] Christoffersen, P. & Jacobs, K. The importance of the loss function in option pricing. *J. Financ. Econ.* **72** (2001).

[23] Tang J (2006). Using bayesian decision for ontology mapping. Web Seman. Sci. Serv. Agents World Wide Web.

[24] Wu, H. *et al.* Multi-class text classification model based on weighted word vector and bilstm-attention optimization. In *International Conference on Intelligent Computing*, 393–400 (Springer, 2021).

[25] Onan A, Toçoğlu MA (2021). A term weighted neural language model and stacked bidirectional lstm based framework for sarcasm identification. IEEE Access.

[26] Sengar, N., Singh, A. & Yadav, V. Classification of documents using bidirectional long short-term memory recurrent neural network. In *Soft Computing and Signal Processing*, 149–156 (Springer, 2021).

[27] Abuqran, S. Arabic multi-topic labelling using bidirectional long short-term memory. In *2021 12th International Conference on Information and Communication Systems (ICICS)*, 492–494 (IEEE, 2021).

[28] Ashburner M (2000). Gene ontology: Tool for the unification of biology. Nat. Genet..

[29] Ye, J. *et al.* Wego 2.0: A web tool for analyzing and plotting go annotations, 2018 update. *Nucleic Acids Res.* **46** (2018). 10.1093/nar/gky400 PMC6030983 29788377

[30] Zhao Y (2020). A literature review of gene function prediction by modeling gene ontology. Front. Genet..

[31] Silla CN, Freitas AA (2011). A survey of hierarchical classification across different application domains. Data Min. Knowl. Discov..

[32] Ramírez-Corona M, Sucar LE, Morales EF (2016). Hierarchical multilabel classification based on path evaluation. Int. J. Approx. Reason..

[33] Liangxi C, Hongfei L, Yuncui H, Jian W, Zhihao Y (2013). Gene function prediction based on the gene ontology hierarchical structure. Plos One.

[34] Wang B, Hu X, Li P, Philip SY (2021). Cognitive structure learning model for hierarchical multi-label text classification. Knowl.-Based Syst..

[35] Valentini G (2015). True path rule hierarchical ensembles for genome-wide gene function prediction. IEEE ACM T. Comput. Bi..

[36] Bi, W. & Kwok, J. T. Hierarchical multilabel classification with minimum bayes risk. In,. IEEE 12th Int. *Conf. on Data Mining* **101–110**, 2012. 10.1109/ICDM.2012.42 (2012).

[37] Blockeel, H., Schietgat, L., Struyf, J., Džeroski, S. & Clare, A. Decision trees for hierarchical multilabel classification: A case study in functional genomics. In *European Conference on Principles of Data Mining and Knowledge Discovery*, 18–29 (Springer, 2006).

[38] Chang C-C, Lin C-J (2011). LIBSVM: A library for support vector machines. ACM Trans. Intell. Syst. Technol..

[39] da Silva, L. V. & Cerri, R. Feature selection for hierarchical multi-label classification. In *IDA*, 196–208 (2021).

[40] Chen B, Hu J (2012). Hierarchical multi-label classification based on over-sampling and hierarchy constraint for gene function prediction. IEEJ Trans. Electr. Electron. Eng..

